# The Influence of Parent- and Adult Child-level Factors on Intergenerational Relationship Quality: A Study of Chinese Families with Multiple Children in Hong Kong

**DOI:** 10.1007/s10823-022-09467-x

**Published:** 2023-01-24

**Authors:** Chang Liu, Xue Bai

**Affiliations:** Centre for Gerontology and Family Studies, Department of Applied Social Sciences, The Hong Kong Polytechnic University, Hung Hom, Hong Kong, China

**Keywords:** hierarchical linear modeling, intergenerational relationship quality, older adults

## Abstract

Intergenerational relationships have become increasingly crucial for maintaining well-being in aging families. Under a changing sociocultural background, families in Hong Kong increasingly exhibit diverse intergenerational relationships and functioning. Focusing on families with mutiple children, this study investigated how the characteristics of parents and their adult children jointly affect different domains of intergenerational relationship quality. A two-stage stratified random sampling design was adopted. Face-to-face questionnaire interviews were conducted between November 2016 and March 2017 with 1,001 Hong Kong residents aged ≥ 50 years. Data of 612 parents and 1,745 adult children were included for analysis. Hierarchical linear modeling was performed to examine child- and parent-level correlates of intergenerational relationship quality. Parents who were female, were married, had higher self-perceived economic status, owned a house, and had fewer depressive symptoms, exhibited higher intergenerational relationship quality. Parents’ age was positively related to affectual closeness, whereas their educational level was negatively related to both affectual closeness and conflict. More favorable intergenerational relationships were reported by aging parents whose adult children were younger, female, and married. Children with higher educational levels exhibited higher levels of both affectual closeness and conflict with their parents. Moreover, affectual closeness was found to be transmitted between generations. The findings can help improve awareness of the factors affecting the different domains of intergenerational relationships, thus informing the development of targeted services and interventions to promote family relationships and well-being.

## Introduction

With increasing longevity, a growing number of parents and children now spend longer shared lifetimes together (Connidis, 2010; Quadagno 2018), thus, high-quality intergenerational relationships have become more crucial. Many studies have reported that positive intergenerational relationships are critical for the health and well-being of both generations throughout their life span (Cong & Silverstein, 2011; Fingerman et al., 2008; Sechrist et al., 2012; Li et al., 2009; Thomas et al., 2017). Therefore, the advancement of intergenerational relationships through comprehensive assessment and accurate identification of facilitators and stressors is becoming increasingly essential.

The multifaceted nature of intergenerational relationships has long been acknowledged in research. The intergenerational solidarity model (Bengtson & Roberts, 1991) highlights the role of family solidarity and proposes six elements that represent interactions and support exchange between parents and children: the extent to which they experience feelings of emotional attachment (affectional dimension), understanding in the relationship (consensual dimension), sharing of norms or expectations of individual obligations to the family (normative dimension), maintenance of contact (associational dimension), provision of instrumental support or sharing of resources (functional dimension), and the existence of opportunities for interaction (structural dimension). In addition to analyzing the positive aspects of intergenerational ties, the conflict perspective draws further attention to the frequent tension and disagreement between parents and their adult children as a natural part of family interactions (Quadagno, 2018). Accordingly, intergenerational conflict was added as the seventh dimension in the intergenerational solidarity model, thereby facilitating a comprehensive understanding of intergenerational relationships (Clarke et al., 1999; Silverstein et al., 1996).

Although the multidimensional character of intergenerational relationship quality is widely recognized, existing studies have typically measured it by using a single or a few select domains. To address this gap in literature, the current study adopted a theory-based multidimensional measure (Bai, 2018) to comprehensively assess intergenerational relationship quality and identify the correlates of the different domains examined.

Although many studies have examined the correlates of intergenerational relationships, most have focused on the characteristics of either the parents or adult children; few individual studies have investigated the joint effects of both parties (Sechrist et al., 2012). According to the concept of “mutual influence” in the family systems theory and “linked lives” in the family life course theory, the lives of significant others are interconnected. When something happens to one member of the family, the lives of other family members also change (Allen & Henderson, 2017). Therefore, the characteristics of both parents and children must be considered during an evaluation of the quality of intergenerational relationships (Allen & Henderson, 2017).

Furthermore, previous studies have seldom focused on families with multiple children to examine the correlates of intergenerational relationships. Some studies have examined a combined sample of multi-child and single-child families, ignoring the fact that multi-child and single-child families can be very different in their systematic properties, such as parenting styles and coordination dynamics (Alidosti et al., 2016; Deane et al., 2016), and consequently, intergenerational relationships. Some studies have found that compared with one-child parents, parents of multiple children reported lower intergenerational relationship quality (e.g., less support exchange and contact) with individual children, however, having more children increased the overall intergenerational relationship quality (Deane et al., 2016; Mickus, 1994). Meanwhile, when examining intergenerational relationships, many studies have combined the data of different children or focused on only one child. Ignoring considerable variations among the data of different siblings can lead to a misleading assumption that parents have uniform experiences of intergenerational relationships with their children, resulting in the loss of critical observations. Instead, the analysis of families with multiple children in the current study examines each parent-child relationship as embedded in the network of other intrafamily relationships. Drawing on this perspective and a dataset that included older parents’ ratings of intergenerational relationship quality with all adult children, hierarchical linear modeling (HLM) was performed to construct a two-level analysis for examining how various factors at the parent and adult child levels are associated with different aspects of intergenerational relationship quality.

Hong Kong provides a suitable context to conduct the present study. Although Hong Kong is deeply influenced by the traditional values of filial piety and emphasis on close family relations, its colonial history and the impacts of Westernization, modernization, and globalization during the past few decades have brought about heterogeneity in intergenerational relationships. The changes in family norms as reflected in marital and reproductive behaviors may also affect intergenerational relationships. The proportion of never-married men aged 25–29 has increased from 82.6% in 2011 to 86.7% in 2021, and the proportion of never-married women has increased from 71.4 to 79.6% (Census and Statistics Department, 2021). Moreover, during recent years, social unrest in Hong Kong has exacerbated conflicts between younger generations and their parents (Shek, 2020), with the younger generations more likely to have unstable employment. Given that families in Hong Kong demonstrate increasingly diverse relationships and functioning under social and demographic changes, obtaining information regarding potential facilitators and stressors that affect the quality of parent-adult child relationships is pertinent.

## Factors Affecting Intergenerational Relationship Quality

Age and gender are particularly important factors affecting family relationships. Studies have indicated that as adult children grow older, their maturational changes are likely to minimize their differences with their parents and increase their tolerance for the remaining disparities, thereby reducing the potential for conflicts and contributing to higher intergenerational closeness and consensus (Bengtson, 1979; Hagestad, 1987). However, the fast-changing sociocultural environment of Hong Kong during the past few decades has led to substantial changes in terms of the values and orientations of younger generations. These changes may threaten the intergenerational consensus and identification supposed to be brought about by maturation.

As children grow older, the intensification of workplace competition and conflicting demands from work and family roles may weaken their ability to provide care for their parents through the maintenance of frequent contact and close relationships (Bai, 2019a; Bai, et al., 2020). Moreover, compared with many Western cultures, traditional Chinese culture places a greater emphasis on sons in terms of elder care (Guo et al., 2013). The higher expectations placed on sons than on daughters may affect the contact frequency, affection, and amount of conflict between parents and their sons (Guo et al., 2013). Therefore, the impact of both the age and gender of adult children and their older parents affecting the parent-child relationship in contemporary Hong Kong families must be investigated.

During the family life course, family members’ transitions, such as marriage or divorce, may also affect dyadic interaction patterns. In China, because of the cultural value of familism, adult children’s marital status can considerably affect intergenerational relationships. Chinese parents may exhibit a lack of acceptance or even feel ashamed of their adult children if they are still single in their 30s. Parents often put pressure on their children to get married, which may result in conflicts and growing distance between two generations. In addition, parents’ divorce is a non-normative transition that may negatively affect adult children’s support and contact frequency (Kalmijn, 2007). However, because of limited evidence in Asian countries, the effect of the marital status of children and parents on intergenerational relationships should be examined.

Furthermore, the non-achievement of adult roles indicative of independence and success (e.g., educational and economic achievements) by adult children can adversely affect the quality of intergenerational relationships (Quadagno, 2018). This phenomenon is particularly observed in Chinese families. Adult children’s education or career achievements can substantially affect their parents’ *mianzi* (prestige) and intergenerational relationships. Moreover, the declining health status of older parents—indicative of the dependency level—is perceived as a crucial predictor of intergenerational relationship quality. With the deterioration of parents’ physical or cognitive health, the need to provide care for parents may intensify pre-existing stress (Pearlin et al., 1981). However, few studies have examined the effect of parents’ socioeconomic status—indicating parents’ dependency level and achievement—on intergenerational relationship quality. Parents’ socioeconomic status is a critical factor because parents’ inability to provide support for children as expected in current society may lead to intergenerational conflict in the family (Shek, 2020). Additional empirical studies are needed to comprehensively examine how the underachievement and dependency of both adult children and parents may affect intergenerational relationships.

Family systems theorists have suggested that the relationship between two family members should be examined as part of a larger system and can be affected by other family relationships, such as the quality of the relationship between parents and grandparents. Bowen (1978) suggested that shared family traits, including thoughts, feelings, and behaviors, lead to similar experiences that can be passed down through generations. In the multigenerational family system, people replicate early interaction patterns and emotional experiences with their own children, thus transmitting the characteristics of intergenerational relationships (Birditt et al., 2012). Although some studies have investigated the transmission of parenting practices and behaviors, only a few have supported the hypothesis regarding the transmission of parent-child relationship quality, especially among Chinese families.

### Conceptual Framework

This study adopted a theory-based multidimensional conceptualization of intergenerational relationships. The conceptual framework was developed based on the main components of the solidarity-conflict model and was validated for Chinese families living in Hong Kong (Bai, 2018). Concerning the correlates of intergenerational relationship quality, Sechrist et al.’s (2012) framework was used to examine both parent- and child-level factors. This framework was based on House’s (1989) perspective of emphasizing the social structural and psychosocial factors of relationship quality and reducing the overemphasis on the problems of family caregiving. The framework intends to focus more on relationships in which the parents are in good health (Sechrist et al., 2012). This aim is consistent with the aim of the current study. Therefore, under the guidance of this framework and with reference to the family systems theory and family life course theory, parent- and adult child-level factors are categorized into four groups: (a) social structural positions, (b) status transitions, (c) independence and achievements, and (d) intergenerational transmission. Specific hypotheses are presented as follows:

### Adult Child-level Hypotheses

H1: Social structural positions (i.e., older age and male sex) are positively correlated with intergenerational relationship quality.

H2: Status transition (i.e., being married) is positively associated with intergenerational relationship quality.

H3: Independence and higher achievements (i.e., being employed and having a higher educational level) are positively associated with intergenerational relationship quality.

### Parent-level Hypotheses

H4: Social structural positions (i.e., older age and female sex) are positively correlated with intergenerational relationship quality.

H5: Status transition (i.e., not being married) is negatively associated with intergenerational relationship quality.

H6: Independence and higher achievements (i.e., better physical and psychological health, higher self-perceived socioeconomic status, higher educational level, and homeownership) are positively correlated with intergenerational relationship quality.

H7: Higher quality of older parents’ relationships with their own parents is positively correlated with the quality of their relationships with their adult children.

## Methods

### Sampling

The data used in the current study were obtained from the research project titled “Intergenerational Relationship and Care Expectations among Chinese Older Adults in Hong Kong”, which is a representative household survey of community-dwelling aging adults. Two-stage stratified sampling was used in the survey to recruit participants who: (a) were aged 50 years or older, (b) were living in Hong Kong, and (c) could speak Cantonese or Mandarin. Finally, a total of 1,001 aging adults were recruited. Additional details of the sampling method have been reported in previous studies (Bai, 2018, 2019b).

### Data Collection

Face-to-face questionnaire interviews were conducted with older parent participants between November 2016 and March 2017. Trained researchers conducted computer-assisted personal interviews to collect data and each interview lasted for approximately 40 minutes. Before visiting the selected households, the researchers sent notification letters to invite participation. Of the 1,966 eligible participants, 234 refused to participate and 731 could not be reached at various times of the day and on different days of the week after more than five visits. Finally, 1,001 participants completed the interviews, yielding a response rate of 50.9%. Because this study intended to focus on multi-child families, data of participants with no children or only one child were excluded. The final sample comprised 612 older parents who provided information related to 1,745 adult children. Ethical approval was obtained before data collection from the Human Subject Ethics Subcommittee of the authors’ affiliated university.

### Measurement

#### Child-level Variables

Age was measured in years. Binary variables included gender (0 = female and 1 = male), employment status (0 = unemployed and 1 = full-time or part-time employed/student), and marital status (0 = unmarried and 1 = married). Educational level was divided into eight categories (from 1 = no formal education to 8 = master’s degree or above).

#### Parent-level Variables

Age, gender, marital status, and educational level were included as parent-level variables. To measure self-perceived economic status, respondents were asked to rate themselves on a hypothetical scale ranging from 1 (lower class) to 5 (upper class). Homeownership was included as a dichotomous variable (0 = tenant and 1 = owner).

Parents’ physical health status was measured based on the number of chronic diseases, while mental health was assessed based on the number of depressive symptoms determined using the five-item Geriatric Depression Scale (Hoyl et al., 1999). Respondents were asked whether they were satisfied with their life, felt upset or helpless, would rather stay at home than go out and try new things, and felt worthless. Total scores ranged from 0 to 5, with a higher score indicating the presence of more depressive symptoms. Cronbach’s alpha for the scale was 0.739 in the current sample.

The quality of respondents’ relationships with their own parents was measured by asking how they perceived the relationship they shared with their parents. The responses ranged from 1 (very poor) to 5 (very good). The quality of relationships with parents was represented by the mean score obtained for the quality of relationship with the father and mother (if available).

#### Dependent Outcome Variable

The intergenerational relationship quality between older parents and adult children was determined based on parents’ responses. Older parents rated their relationship quality with every adult child using the 13-item Intergenerational Relationship Quality Scale for Aging Parents (Bai, 2018), which assesses structural-associational solidarity, affectual closeness, consensual-normative solidarity, and intergenerational conflict. The scope of the scale includes residential proximity, frequency of face-to-face contact and communication by phone, letter, or email, helping children with household chores, how well each respondent gets along with their children, general feelings of closeness with children, the frequency of receiving gifts from children, degrees of similarity in opinions concerning social and political issues and filial responsibilities of elder care, and degrees of tension and strained feelings between the two generations. Respondents rated all items on a 5-point Likert scale. The score range for the structural-associational solidarity subdomain was 4-20 and the total scores of the other three subdomains ranged from 3 to 15. The total score of the scale ranged from 13 to 65, with the scores of the intergenerational conflict subdomain being reverse coded. A higher score indicated a more satisfactory intergenerational relationship quality. In this study, Cronbach’s alpha was 0.786 for overall reliability and 0.762, 0.781, 0.905, and 0.816 for the subdomains of structural-associational solidarity, affectual closeness, consensual-normative solidarity, and intergenerational conflict, respectively.

### Data Analysis

HLM was adopted to estimate the joint effects of parents’ and children’s characteristics on the quality of intergenerational relationships. The unit of analysis in this study is the individual parent-child dyad. Because adult children are grouped within the category of parents, the intergenerational relationship quality of adult children within the same family will be correlated. This correlation violated a core statistical assumption of standard regression methods that all observations should be independent of one another (Raudenbush & Bryk, 2002). If this correlation is ignored, incorrect inferences can result in the false estimation of the effects of both parent- and child-level factors. HLM was used in the data analysis because it can appropriately examine the nested data of multiple siblings and allow child-level predictors to be represented as within-family differences (Silverstein et al., 2012). In this study, child-level models were first computed to assess the relationships between intergenerational relationship quality and child-level variables. One regression equation was calculated with intercepts and slopes for each parent, which was then used in the parent-level analysis to further examine the influence of parent-level factors on intergenerational relationship quality.

The SPSS 20.0 (IBM Corp, 2013) and HLM 7.0 (Raudenbush et al., 2011) software were used for data analysis. Among the 1,001 respondents, those with no children or only one child aged ≥ 18 years (*n* = 389) were excluded from the present study. Finally, data of 612 parents and 1,745 adult children (average number of children = 2.95) were included for analysis. The relationship quality of 1,745 parent-child dyads was determined based on parents’ responses.

To examine the nested relationship of intergenerational relationships within families, data were arranged hierarchically. Level 1 included child-level individual characteristics, while Level 2 included parent-level characteristics. All missing values for Level 2 variables were replaced with the group mean because the percentages of the missing cases were all below 5% (Hair et al., 2010). Descriptive analysis was first separately conducted for child- and parent-level variables. Subsequently, HLM was used to perform a multilevel ordinal regression analysis using both child- and parent-level variables. The quality of intergenerational relationships was assumed to be predicted by the adult children’s age, gender, marital status, educational level, and employment status (Level 1). In addition, the parent-child relationship was predicted to differ according to parents’ age, gender, marital status, educational level, self-perceived economic status, homeownership status, physical health status, depressive symptoms, and relationship with their own parents (Level 2). First, the Level 1 variables were entered into a multilevel model after grand mean centering to determine the significance level of the intercepts. The overall intergenerational relationship and the four subdomains were considered the outcome variables. The regression slope of each outcome variable was then treated as a dependent variable and the grand mean centered Level 2 variables were added into the models to examine if any Level 2 variable was significantly related to the slope.

The fixed effects in the multilevel regression were tested by the ratio of the intercept to the estimate of the standard error. Coefficients, standard errors, *t* ratio values and significance, and intra-class coefficients (ICCs) were calculated. The proportion of variances accounting for the dependent variable from Level 1 and Level 2 was calculated using Snijders and Bosker’s (1999) method.

### Statistical Analysis

Correlations between the child-level characteristics and outcome variables were initially examined. The model for examining intergenerational relationship with all children-level measures is:

*Y*_*ij*_ *= β*_*0j*_ *+ β*_*1*_ (age) *+ β*_*2*_ (gender) *+ β*_*3*_ (marital status) *+ β*_*4*_ (educational level) *+ β*_*5*_ (work status) *+ r*_*ij*_.

where *i* and *j* indicate the effects of the children’s and parents’ characteristics, respectively. The distribution of *r*_*ij*_ was assumed to be random normal with a mean of 0 and a variance of σ^2^.

To examine the effect of parents’ characteristics on intergenerational relationships, we modeled the effects of parent-level variables on the intercept from the previous model as follows:

*β*_*0j*_ *= γ*_*00*_ *+ γ*_*01*_ (age) *+ γ*_*02*_ (gender) *+ γ*_*03*_ (marital status) *+ γ*_*04*_ (educational level) *+ γ*_*05*_ (self-perceived economic status) *+ γ*_*06*_ (homeownership) *+ γ*_*07*_ (number of chronic diseases) *+ γ*_*08*_ (depressive symptoms) *+ γ*_*09*_ (relationship with parents) *+ u*_*0j*_.

In this model, *j* indicates the effects of parents’ characteristics, *β*_*0j*_ is the intercept term from the child-level equation, and *u*_*0j*_ is a parent-level disturbance that is assumed to be normally distributed with a mean of 0 and a variance of τ.

## Results

As shown in Table 1, the average age of the respondents was 69.384 years. Of all the respondents, 42.5% were men and 57.5% were women. In terms of marital status, 60.5% of the respondents were married and living with their spouse, and the remaining respondents were separated, divorced, or widowed. The mean (standard deviation [SD]) of the educational level of the respondents was 2.334 (1.177) between primary and junior high school. The mean (SD) of self-perceived economic status was 2.961 (0.568). The majority of respondents (69.8%) lived in rented houses, and only 30.2% of respondents owned private or subsidized houses. The average number of chronic diseases was 1.119, and the mean of depressive symptoms was 1.041, indicating relatively satisfactory health conditions of respondents, both physically and mentally. In addition, the respondents reported having considerably high-quality relationships with their own parents (4.063).

**Table 1:** Descriptive statistics of study variables

Variable Name	Mean/Frequencies	SD/%	Range
**Level 1: Child characteristics (*n*** **= 1,745)**			
Age	40.814	11.663	19–73
Gender (male)	877	50.3%	
Marital status (married)	615	35.2%	
Educational level	4.222	1.133	1–7
Junior secondary education and below	306	17.7%	
Senior secondary education	1,106	64.1%	
Above senior secondary education	314	18.2%	
Working status (student/employed)	1,432	84.8%	
**Level 2: Parental characteristics (*n*** **= 612)**			
Age	69.384	10.814	50–102
Gender (male)	260	42.5%	
Marital status (married)	370	60.5%	
Educational level	2.334	1.177	1–8
Primary education and below	413	67.5%	
Junior secondary education	111	18.1%	
Above Junior secondary education	88	14.4%	
Self-perceived economic status	2.961	0.568	1–5
Homeownership (homeowner)	185	30.2%	
Number of chronic diseases	1.119	1.129	2–10
Depressive symptoms	1.041	1.373	0–5
Relationship with parents	4.063	0.622	2–5
**Outcome variables**			
Intergenerational relationship quality	43.898	7.146	17–62
Affectual closeness	11.025	2.370	3–15
Structural-associational solidarity	12.393	3.868	4–20
Consensual-normative solidarity	8.273	2.467	3–15
Intergenerational conflict	12.209	2.339	3–15

The total number of valid adult children in this study was 1,745, and their average age was 40.814 years. Among the adult children, 50.3% were sons and 35.2% were married. On average, most of them had studied up to senior high school (4.222). The majority of the adult children were employed or students (84.8%).

In terms of intergenerational relationship quality, the mean (SD) of the total score and four subdomains (affectual closeness, structural-associational solidarity, consensual-normative solidarity, and intergenerational conflict) was 43.898 (7.146), 11.025 (2.370), 12.393 (3.868), 8.273 (2.467), and 12.209 (2.339), respectively.

Table 2 lists the results of the multilevel ordinal regression models. The ICCs ranged from 0.515 (structural-associational solidarity) to 0.915 (intergenerational conflict), indicating significant variance (*p* < 0.001) across families in terms of the overall intergenerational relationship and four subdomains.

**Table 2:** Results of multilevel linear models for overall intergenerational relationship and subdomains

	Intergenerational relationship		Affectual closeness		Structural-associational solidarity	
	Coefficient (SE)	t ratio	Coefficient (SE)	t ratio	Coefficient (SE)	t ratio
**Level 1: child characteristics**						
Age	-0.092 (0.026)	-3.483***	-0.006 (0.008)	-0.776	-0.093 (0.015)	-6.163***
Gender	-0.629 (0.216)	-2.907**	-0.320 (0.061)	-5.195***	0.215 (0.132)	1.630
Marital status	3.105 (0.308)	10.082***	0.218 (0.088)	2.477*	2.919 (0.209)	13.994***
Educational level	0.169 (0.151)	1.114	0.201 (0.049)	4.124***	-0.149 (0.076)	-1.953
Work status	0.275 (0.445)	0.617	0.242 (0.138)	1.751	-0.136 (0.238)	-0.569
**Level 2: parental characteristics**						
Age	0.033 (0.033)	0.987	0.028 (0.010)	2.679**	-0.004 (0.017)	-0.208
Gender	-2.372 (0.477)	-4.966***	-0.781 (0.170)	-4.584***	-0.981 (0.243)	-4.045***
Marital status	1.129 (0.505)	2.233*	0.238 (0.174)	1.366	0.441 (0.255)	1.732
Education level	-0.280 (0.244)	-1.149	-0.189 (0.076)	-2.473*	-0.061 (0.103)	-0.598
Self-perceived economic status	1.132 (0.432)	2.621***	0.386 (0.147)	2.618**	0.244 (0.206)	1.185
Homeownership	1.076 (0.465)	2.315*	0.082 (0.169)	0.483	0.367 (0.236)	1.551
Number of Chronic Disease	0.009 (0.221)	0.042	0.141 (0.076)	1.841	0.036 (0.096)	0.383
Depressive symptoms	-1.504 (0.194)	-7.754***	-0.529 (0.065)	-8.184***	-0.352 (0.089)	-3.941***
Relationship with parents	0.627 (0.358)	1.746	0.810 (0.126)	6.411***	-0.006 (0.185)	-0.032
Intercept	43.956 (0.217)	202.041***	10.982 (0.079)	139.006***	12.483 (0.109)	114.726***
Intra-class correlation (ICC)	0.680		0.794		0.515	
*R*^*2*^ at Level 1	0.382		0.329		0.413	
*R*^*2*^ at Level 2	0.433		0.349		0.503	

*Note.* *** *p* < 0.001, ** *p* < 0.01, * *p* < 0.05

**Table 2:** (Continuous) Results of multilevel linear models for overall intergenerational relationship and subdomains

	Consensual-normative solidarity		Intergenerational conflict	
	Coefficient (SE)	t ratio	Coefficient (SE)	t ratio
**Level 1: child characteristics**				
Age	-0.013 (0.008)	-1.549	0.012 (0.006)	2.083*
Gender	-0.451 (0.071)	-6.367***	-0.069 (0.041)	-1.671
Marital status	0.167 (0.085)	1.947	-0.179 (0.060)	-2.984**
Educational level	0.028 (0.056)	0.500	0.097 (0.032)	2.985**
Work status	0.109 (0.152)	0.717	0.081 (0.071)	1.130
**Level 2: parental characteristics**				
Age	0.020 (0.013)	1.578	-0.001 (0.011)	-0.116
Gender	-0.338 (0.207)	-1.634	-0.210 (0.202)	-1.043
Marital status	0.516 (0.213)	2.424*	-0.064 (0.206)	-0.309
Educational level	0.173 (0.087)	1.981*	-0.219 (0.092)	-2.373*
Self-perceived economic status	0.169 (0.173)	0.978	0.394 (0.173)	2.280*
Homeownership	0.323 (0.184)	1.753	0.358 (0.202)	1.776
Number of Chronic Disease	-0.147 (0.091)	-1.611	0.020 (0.089)	0.225
Depressive symptoms	-0.291 (0.078)	-3.735***	-0.328 (0.075)	-4.362***
Relationship with parents	-0.181 (0.148)	-1.219	-0.029 (0.149)	-0.192
Constant	8.329 (0.091)	91.873***	12.143 (0.090)	134.208***
Intra-class correlation (ICC)	0.834		0.915	
*R*^*2*^ at Level 1	0.189		0.124	
*R*^*2*^ at Level 2	0.182		0.127	

*Note.* *** *p* < 0.001, ** *p* < 0.01, * *p* < 0.05

In the overall model, children’s age (B = − 0.092 [0.026], *p* < 0.001), gender (B = − 0.629 [0.216], *p* < 0.01), and marital status (B = 3.105 [0.308], *p* < 0.001) exhibited significant effects on the overall intergenerational relationship quality reported by parents. Of the parent-level variables, gender (B = − 2.372 [0.477], *p* < 0.001), marital status (B = 1.129 [0.505], *p* < 0.05), self-perceived economic status (B = 1.132 [0.432], *p* < 0.001), homeownership status (B = 1.076 [0.465], *p* < 0.05), and depressive symptoms (B = − 1.504 [0.194], *p* < 0.001) were significantly associated with the average score of intergenerational relationship quality. The intergenerational relationship quality was higher when children were younger, female, and married and when parents were female, were married, owned a home, had higher self-perceived economic status, and had fewer depressive symptoms. Overall, potential predictors explained 38.2% and 43.3% of the variance in intergenerational relationship quality for Level 1 and Level 2 variables, respectively.

The Level 1 and Level 2 variables exerted different effects on the four subdomains of intergenerational relationship quality. Children’s gender (B = − 0.320 [0.061], *p* < 0.001), marital status (B = 0.218 [0.088], *p* < 0.05), and educational level (B = 0.201 [0.049], *p* < 0.001) exerted significant effects on affectual closeness. Parents’ age (B = 0.028 [0.010], *p* < 0.01), gender (B = − 0.781 [0.170], *p* < 0.001), educational level (B = − 0.189 [0.076], *p* < 0.05), self-perceived economic status (B = 0.386 [0.147], *p* < 0.01), depressive symptoms (B = − 0.529 [0.065], *p* < 0.001), and relationship with their own parents (B = 0.810 [0.126], *p* < 0.001) were associated with affectual closeness with their children. The Level 1 and Level 2 variables explained a total of 32.9% and 34.9% of the variance in affectual closeness, respectively.

In terms of structural-associational solidarity, children’s age (B = − 0.093 [0.015], *p* < 0.001) and marital status (B = 2.919 [0.209], *p* < 0.001) affected parents’ reported structural-associational solidarity. Parents’ gender (B = − 0.981 [0.243], *p* < 0.001) and depressive symptoms (B = − 0.352 [0.089], *p* < 0.001) were negatively associated with structural-associational solidarity. The Level 1 and Level 2 variables explained 41.3% and 50.3% of the variance in structural-associational solidarity, respectively.

In terms of consensual-normative solidarity, the Level 1 and Level 2 variables explained 18.9% and 18.2% of the variance, respectively. Among Level 1 variables, children’s gender (B = − 0.451 [0.071], *p* < 0.001) was negatively associated with intergenerational consensus. Among Level 2 variables, parents’ marital status (B = 0.516 [0.213], *p* < 0.05) and educational level (B = 0.173 [0.087], *p* < 0.05) were positively associated with consensual-normative solidarity, whereas the number of depressive symptoms (B = − 0.291 [0.078], *p* < 0.001) was negatively associated with consensual-normative solidarity.

The Level 1 and Level 2 variables explained 12.4% and 12.7% of the variance in intergenerational conflict, respectively. Children’s age (B = 0.012 [0.006], *p* < 0.05) and educational level (B = 0.097 [0.032], *p* < 0.01), as well as parents’ self-perceived socioeconomic status (B = 0.394 [0.173], *p* < 0.05) were positively associated with intergenerational conflict, whereas children’s marital status (B = − 0.179 [0.060], *p* < 0.01), and parents’ educational level (B = − 0.219 [0.092], *p* < 0.05) and depressive symptoms (B = − 0.328 [0.075], *p* < 0.001) were negatively associated with intergenerational conflict.

## Discussion and Implications

Although the quality of intergenerational relationships has long been a topic of interest in aging and family research, limited studies have focused on the intergenerational relationships between older parents and individual adult children within the whole family context. To the best of our knowledge, the present study is among the first to investigate the multilevel correlates of the multiple domains of intergenerational relationship quality by focusing on families with multiple children in Hong Kong. The findings of this study can improve understanding regarding all aspects of interactions in contemporary multi-child aging families in Hong Kong and are crucial for developing targeted services and interventions for different groups of parents and adult children.

In the current study, adult children’s older age was significantly correlated with a decreased overall quality of intergenerational relationships, a lower level of structural-associational solidarity, and a higher level of intergenerational conflict. These findings reject the first half of H1 and are inconsistent with the results of previous studies that have reported that older adult children tend to have a less conflictual relationship with their parents (Birditt et al., 2009) and that older children are likely to be a crucial source of intergenerational support (Suitor & Pillemer, 2007). By contrast, these findings, to some extent, reflect a reality that is heavily influenced by globalization and modernization; thus, younger generations may no longer follow their parents’ lifestyles or cling strongly to traditional values (Long & Feng, 2007; Zhu & Ouyang, 2015). Therefore, the older age of adult children does not necessarily correspond to them being more similar to their parents in terms of social roles and behaviors. Disparities between two generations are even more pronounced as adult children become older, leading to a higher level of intergenerational conflict. Moreover, as adult children become older, they are more likely to experience increased pressures in their daily life including taking care of their own children. This, in turn, may reduce their availability for their parents, thereby reducing the contact frequency and structural-associational solidarity between the two generations and leading to more sources of conflict.

The second half of H1 is also not supported because daughters were found to have a more favorable relationship with their parents, higher affectual closeness, and stronger intergenerational consensus. In contrast to the findings of previous studies conducted in Chinese societies (Bian et al., 1998; Guo et al., 2013), the strong preference for sons in Chinese families was not reflected in the current study. Apart from gender differences in the motivation to maintain intergenerational ties, sons likely maintain relationships with their parents in other forms, such as by providing instrumental or financial support that may not be fully captured in the current study. Additional qualitative studies should be conducted to explore the characteristics of intergenerational relationships between sons and parents and to investigate specific challenges encountered by sons and older adult children in their efforts to maintain a harmonious relationship with their parents among aging families in Hong Kong. In addition, targeted services should be provided for these two groups of adult children.

Consistent with H2, married adult children were observed to have higher overall intergenerational relationship quality and higher levels of affectual closeness and structural-associational solidarity, and a lower level of intergenerational conflict with their parents. These findings are consistent with those of previous studies (e.g. Silverstein et al., 2010; Umberson, 1992). Regarding H3, educational level was found to be positively associated with affectual closeness and intergenerational conflict, whereas employment status was not correlated with any domain of relationship quality. In the current study, Chinese parents’ high expectations of adult children receiving higher education may contribute to the simultaneous existence of affectionate and conflictual relationships. A possible explanation is that adult children with a higher educational level may face higher expectations in terms of their personal achievements and responsibilities toward their families. Such high expectations may result in an affectionate bond, however, may also cause conflicts when the expectations cannot be met. This phenomenon in particular should be investigated in future studies.

The roles of parents’ gender, age, and marital status were generally consistent with H4 and H5. For example, mothers reported higher intergenerational relationship quality and higher levels of affectual closeness and structural-associational solidarity with their adult children, which is in agreement with the findings of previous studies (Birditt et al., 2012; Silverstein et al., 2010; Ward, 2008). Together with previous findings, the current study provides supporting evidence regarding the kin-keeping role of mothers and daughters, as suggested by Kalmijn (2005), who examined a sample from Hong Kong. Moreover, parents’ older age was associated with a higher level of affectual closeness, and married parents exhibited higher intergenerational relationship quality and a higher level of consensual-normative solidarity. Married parents were more likely to enjoy higher overall intergenerational relationship quality and a higher level of consensual-normative solidarity compared with their unmarried counterparts. Thus, during the development of social services and intervention programs targeting the improvement of family relationships, special attention should be paid to improving the relationships between fathers and adult children and between children and unmarried parents.

H6 was partially supported. Parents’ physical health was found to exert no effect on any domain of intergenerational relationship quality, which is inconsistent with the results of previous studies (e.g., Cicirelli, 2000). This finding may be attributed to the relatively good physical health of the participants in the current study, as reflected by their average number of chronic diseases. Thus, they might not have required considerable instrumental support from their children. Furthermore, this finding implies that the adult children in this study may not yet have assumed the role of caregiver. Therefore, the negative or positive effects of physical health on caregiving did not emerge in this study. The physical health status neither intensified strains nor increased closeness in parent-adult child relationships. By contrast, older parents’ psychological health was a crucial factor significantly correlated with all domains of intergenerational relationship quality. These findings are consistent with those of previous studies that have reported an association of a higher number of depressive symptoms with a lower level of affectual closeness (Ha, 2009) and a decreased frequency of contact (Tosi & Grundy, 2019). Additional longitudinal and qualitative studies should be conducted to determine the mechanism through which parents’ psychological health affects their interactions and contact with their adult children. In addition, service providers should pay more attention to the mental health of aging adults and develop targeted services to help them maintain a favorable psychological health status.

In the present study, a higher level of parents’ self-perceived socioeconomic status was found to be associated with higher overall intergenerational relationship quality, a higher level of affectual closeness, and a higher level of intergenerational conflict. Moreover, parents’ homeownership status was associated with a higher overall quality of their relationship with their adult children. This is possibly because parents with higher socioeconomic status are generally more likely to be providers than recipients of support (Grundy, 2005), which is helpful to maintain a close intergenerational relationship. However, conflicts are also likely to arise over parental control of high-socioeconomic status parents. Moreover, parents’ higher educational level was negatively correlated with both affectual closeness and intergenerational conflict but positively correlated with consensual-normative solidarity. This finding suggests that compared with children’s educational level, parents’ educational level plays a completely different role in affecting intergenerational relationship quality, possibly because parents with a higher educational level are more likely to embrace non-traditional values and reach a consensus with younger generations. Moreover, parents with a higher educational level may have more interests and friends outside of the family and prefer independence (Bian et al., 1998), which may reduce their conflict with their adult children and lead to less demand for emotional closeness.

Regarding H7, the findings of the current study provide support for the intergenerational transmission of relationship quality, which has seldom been examined in previous studies. Even after the other characteristics of both generations were controlled for, participants who reported a more favorable relationship with their own parents also exhibited a significantly higher level of affectual closeness with their adult children. This finding accords with similar studies conducted in the United States and Europe (Birditt et al., 2012; Hank et al., 2017; Savelieva et al., 2017). The findings of the current study provide empirical support for relevant theories among Asian families. However, determining the quality of the relationships between grandparents and parents in the current study could capture only a single domain of the relationship (i.e., affectual closeness). Future studies should examine the transmission of multiple domains of intergenerational relationship quality and elucidate the potential mechanism through which intergenerational relationship quality is transmitted in Chinese families. Such studies can help increase the awareness of policymakers and family service providers to develop policies and programs that aim at not only improving dyadic relationships but also promoting better relationships across multiple generations in the whole family system.

### Limitations

Although the present study reveals important findings, it has several limitations that should be acknowledged. First, because this was a cross-sectional study, the ability to establish causality was limited. Additional longitudinal studies are necessary to clarify the relationships between multiple correlates and relationship quality, and qualitative studies can help explain the causality between variables. Second, only the perspectives of older parents were collected in this study. Different results may have been obtained if the views of adult children were examined because compared with younger adults, older generations tend to report better and closer relationships with younger generations (Kim & Stokes, 2019). Additional studies should collect and examine data from both parties. Third, this study only focused on the correlates of intergenerational relationships in multi-child families. Future studies should investigate the topic in single-child families and compare the differences between multi-child and single-child families for a more comprehensive understanding of the topic. Last, because of limitations in the measurement of correlates, the current study could not examine an exhaustive list of potential influential factors. Future studies should consider investigating a wider range of predictors of multidimensional intergenerational relationship quality, such as adult children’s parental status and health status, as well as other social-level factors.

### Conclusion

Focusing on multi-child families, this study used HLM to investigate how characteristics at the parent- and adult child-levels jointly affected the different domains of intergenerational relationship quality in a representative sample of aging parents in Hong Kong. The results revealed how older parents’ and adult children’s social structural positions, status transition, independence, and achievements impact intergenerational relationship quality and provide support for the transmission of intergenerational relationship quality across generations. The findings can improve awareness regarding factors that may affect the different domains of intergenerational relationships, thus informing the development of targeted services and interventions for different groups of children and parents to enhance family relationships and overall well-being.

## Data Availability

The data that support the findings of this study are available from the corresponding author upon reasonable request.
